# Accelerated high fluence photoactivated chromophore for infectious keratitis—corneal cross-linking (PACK-CXL) at the slit lamp: a pilot study

**DOI:** 10.3389/fphar.2023.1229095

**Published:** 2023-09-07

**Authors:** Hagar Olshaker, Asaf Achiron, Alexander Chorny, Farhad Hafezi, Tal Yahalomi, Assaf Kratz, Erez Tsumi, Nan-Ji Lu, Boris Knyazer

**Affiliations:** ^1^ Tel Aviv Sourasky Medical Center, Sackler School of Medicine, Tel Aviv University, Tel Aviv, Israel; ^2^ Department of Ophthalmology, Soroka University Medical Center, Faculty of Health Sciences, Ben-Gurion University of the Negev, Be’er Sheva, Israel; ^3^ Center for Applied Biotechnology and Molecular Medicine (CABMM), University of Zurich, Zurich, Switzerland; ^4^ ELZA Institute, Dietikon, Switzerland; ^5^ Faculty of Medicine, University of Geneva, Geneva, Switzerland; ^6^ Department of Ophthalmology, University of Southern California Roski Eye Institute, University of Southern California, Los Angeles, Los Angeles, CA, United States; ^7^ School of Ophthalmology and Optometry, Wenzhou Medical University, Wenzhou, China; ^8^ Department of Ophthalmology, Samson Assuta Ashdod, Faculty of Health Sciences, Ben-Gurion University of the Negev, Be’er Sheva, Israel

**Keywords:** corneal cross-linking, infectious keratitis, PACK-CXL, high fluence, hypo-osmolar riboflavin, outcome

## Abstract

**Introduction:** Photoactivated Chromophore for Infectious Keratitis-Corneal Cross-Linking (PACK-CXL) has garnered substantial interest among researchers and ophthalmologists due to its high promise as a potential treatment for infectious keratitis. The aim of this study is to evaluate the efficacy and safety of high fluence PACK-CXL, using 10.0 J/cm^2^ (30 mW/cm^2^, 5 min, and 33 s) at the slit lamp.

**Methods:** This prospective interventional, nonrandomized cohort study included 20 eyes of 20 patients with bacterial, fungal, or mixed origin keratitis who underwent high fluence PACK-CXL treatment as an adjunct therapy to conventional antimicrobial therapy per American Academy of Ophthalmology treatment guidelines. The re-epithelization time was recorded, and corneal endothelial cell density was counted before and after treatment.

**Results:** The average re-epithelization time was 8.2 ± 2.8 days (range 3–14 days). After PACK-CXL treatment, eight patients (40%) were directly discharged, while the remained patients stayed in the hospital for an average of 5.6 ± 3.5 days. No eyes required keratoplasty. Endothelial cell density counts before and after the PACK-CXL procedure were 2,562.1 ± 397.3, and 2,564.8 ± 404.5 cells/mm^2^, respectively (*p* = 0.96).

**Conclusion:** although it was not a randomized control trial, we conclude that high fluence PACK-CXL as an adjuvant therapy is safe with no complications observed, and efficient as time to re-epithelization was less than 14 days for all patients and no patients underwent tectonic keratoplasties. Further research is needed to compare it to the current standard of care.

## 1 Introduction

Infectious keratitis is a sight-threatening infection of the cornea. Several pathological organisms can cause infectious keratitis, and its major risk factors include contact lens wear, trauma, and eyelid and ocular surface disorders (Al-Mujaini et al., 2009; Ting et al., 2022). Prompt treatment with topical broad-spectrum antimicrobial eye drops is the standard of care (Lin et al., 2018), and additional interventions such as corneal debridement may be used in some cases. Once the pathogen has been identified, more targeted therapy can be used according to the causative pathogen’s antimicrobial drug sensitivity profile (Barac et al., 2022). However, even with prompt treatment, outcomes are not always favorable due to various factors such as antibiotic resistance, low bacterial isolation rates, limited corneal drug penetration, plus several other predisposing risk factors (Knyazer et al., 2020).

Corneal cross-linking (CXL) is a minimally invasive procedure used to strengthen the cornea and today it is widely utilized for the treatment of ectatic corneal diseases. It is based on a photochemical reaction caused by the combination of a photoactivated chromophore (riboflavin, most commonly) and a photoactivation wavelength of light (in the case of riboflavin, is ultraviolet-A light at around 365–370 nm) (Price and Price, 2016; Kling et al., 2020).

In addition to its mechanical benefits in strengthening the cornea, photoactivation of chromophore has been shown to act like a disinfectant by various mechanisms (Hafezi and Bradley Randleman, 2014). As such, CXL was proposed as a novel treatment for infectious keratitis, termed “photoactivated chromophore for infectious keratitis—corneal cross-linking”, or PACK-CXL. It was first introduced as an adjuvant treatment to standard-of-care antimicrobial therapy (Idrus et al., 2019; Ting et al., 2019; Gulias-Cañizo et al., 2020; Knyazer et al., 2020; Achiron et al., 2021a; Saini et al., 2022), then later as a stand-alone treatment (Hafezi et al., 2022a; Kling et al., 2020; Knyazer et al., 2020; Hafezi et al., 2021).

Historically, most PACK-CXL treatments were given using the Dresden protocol settings developed for keratoconus, i.e., irradiation settings of 3 mW/cm^2^ for 30 min to deliver a UV fluence of 5.4 J/cm^2^ (Koppen et al., 2009; Müller et al., 2012; Price et al., 2012; Bamdad et al., 2015; Prajna et al., 2020). Accelerated protocols, employing a power density of 30 mW/cm^2^ for a duration of 3 min (equivalent to a total energy dose of 5.4 J/cm^2^), have also been implemented (Richoz et al., 2014; Achiron et al., 2021b). However, because corneal ulcers are opaque, the depth of microbial invasion can be considerably deeper than the approximate 330 µm depth of cross-linking effect achieved by the standard 5.4 J/cm^2^ applied in the Dresden protocol cross-linking (Hafezi et al., 2020). Hence, we believe that the pathogen-killing efficacy of PACK-CXL can be improved by increasing the total irradiation fluence. In a recent *in vitro* study, Kling et al. (Kling et al., 2020) tested the bacteria-killing effect of irradiation different intensities (ranging from 3 to 18 mW/cm^2^) and different fluences (ranging from 5.4 to 27 J/cm^2^) on six different bacterial strains. Their study showed that for *Staphylococcus aureus*, the ratio of bacterial killing was 50% at 5.4 J/cm^2^ and increased to 92% at 10.8 J/cm^2^ and 100% at 16.2 J/cm^2^ and above (Kling et al., 2020). More *in vitro* studies support this result (Lu et al., 2023). *In vivo* studies examining high fluences on infectious keratitis, however, are scarce (Nateghi Pettersson et al., 2019; Knyazer et al., 2021; Hafezi et al., 2022a; Awad et al., 2022; Hafezi et al., 2022b; Marrie et al., 2023), mostly on animal models or human case reports. Our first aim is to show the therapeutic benefit of high fluence PACK-CXL in a case series.

UV irradiation above a certain threshold can damage the corneal endothelium. Although riboflavin shields the endothelium from UV energy, it gets consumed during the process, so there is a limit to the amount of UV energy that can be safely delivered during corneal cross-linking procedures. The safety of high fluence UV irradiation protocols was demonstrated by Mazzotta et al. (2016) who studied them in patients with keratoconus, where they found that delivering high fluences (7.2, 10, and 15 J/cm^2^) was as safe as both conventional (Dresden protocol) and accelerated CXL procedures. Our second aim in this study is to examine the safety of high fluence CXL protocols by assessing complications such as corneal melting of corneal perforation.

Although in practice PACK-CXL is not yet as widely adopted or established as traditional corneal cross-linking, this innovative approach holds great potential in augmenting the standard treatment options for infectious keratitis, leading to improved outcomes and preserving visual acuity for affected individuals.

The data presented here is, to our knowledge, the first trial testing the therapeutic effect of PACK-CXL at the slit lamp on patients with infectious keratitis using a high fluence of 10.0 J/cm^2^ rather than the typical fluence of 5.4 J/cm^2^.

## 2 Materials and methods

This prospective interventional, nonrandomized cohort study was approved by the Institutional Review Board (IRB) of the Ben-Gurion University of the Negev, Israel (0486-20-SOR), and adhered to the tenets of the Declaration of Helsinki. All patients gave written informed consent prior to inclusion into this study.

### 2.1 Patients inclusion and exclusion criteria

Patients admitted to the emergency room of the Department of Ophthalmology at Soroka University Medical Center, Beer-Sheva, Israel (SUMC), between March 2021 and February 2022, were enrolled. The inclusion criteria were acute corneal infiltrate/ulcer of suspected bacterial, fungal or mixed (bacterial and fungal) origin, a maximal length of 6 mm, maximal infiltrate/ulcer depth of 350 µm as determined by anterior segment optical coherence tomography (AS-OCT, Spectralis, Heidelberg, Franklin, United States), and an existing epithelial defect. The exclusion criteria were suspicion of a sterile, viral or *Acanthamoeba* keratitis, descemetocele or perforated cornea, pregnancy or breastfeeding, monocular patient, systemic steroidal treatment or other immunosuppression/immunocompromised condition, active corneal herpetic disease, and diagnosed eczema (due to their frequent ocular co-morbidities and concurrent use of steroidal treatments, as these factors could potentially cause bias in the results).

### 2.2 Initial examination and treatment

The same corneal consultant (BK) was in charge of managing all patients and decided to perform PACK-CXL treatment. The initial examination included the slit lamp evaluation and anterior segment photography, AS-OCT, specular microscopy endothelial cell count (EM-3000 specular microscope, Tomey GmbH, Germany), and visual acuity. Corneal scrapings for Gram staining, bacteriologic, and fungal study were taken, including blood, chocolate, and Sabouraud agar. All patients were treated based on the American Academy of Ophthalmology (AAO) guidelines for infectious keratitis before high fluence PACK-CXL (Lin et al., 2018). Some patients were immediately referred to adjuvant PACK-CXL treatment and some were given a trial of topical treatment first, according to ulcer severity. Following PACK-CXL, patients continued to receive the same topical medication regimen they were given prior to treatment, a combination of antimicrobial topical medications according to the specific pathogen identified, and were closely monitored.

### 2.3 PACK-CXL technique

To prevent any interference with riboflavin activity, any patient chosen for the procedure had to wait at least 24 h following fluorescein staining. PACK-CXL was performed in a sterile setting under topical anaesthesia with 0.4% oxybuprocaine hydrochloride drops (Localin, Fischer Pharmaceuticals, Tel Aviv, Israel). A lid speculum was positioned to ensure the eye’s stability and secure placement. Corneal cross linking as the slit lamp was done before and was previously elaborated (Hafezi et al., 2021). In short, corneal epithelium debridement was made circumferentially using a hockey knife around the borders of the infected ulcer. The patient was then moved to a reclining chair, and hypo-osmolar 0.1% riboflavin solution (Ribo-Ker; EMAGine SA) was instilled enough to cover the whole cornea, every 2 min for 20 min. The patient then returned to the slit lamp stool, and the cornea was irradiated by an ultraviolet-A (UV-A) light at 365 nm using a slit lamp-mounted CXL device (C-eye, EMAGine AG, Zug, Switzerland) at an intensity of 30 mW/cm^2^ for 5 min and 33 s (fluence: 10.0 J/cm^2^) (Knyazer et al., 2021). Table 1 describes the PACK-CXL method.

**TABLE 1:** PACK-CXL procedure: cross-linking device technical settings, irradiation protocols, and riboflavin used.

Parameter	Variable
Treatment target	Infectious keratitis treatment
Fluence (total; J/cm^2^)	10.0
Soak Time (minutes)	20
Intensity (mW/cm^2^)	30
Treatment time (minutes)	5.5
Epithelium status	Off
Chromophore	Ribo-Ker 0.1%; EMAGine SA
Light Source	C-eye
Irradiation mode	Continuous
Protocol modification	Abrasion 1 mm around the border corneal infiltrate/ulcer; treatment 2 mm around the border corneal infiltrate/ulcer

### 2.4 Statistical analysis

Statistical analyses were performed using SPSS software (Version 25, IBM, Chicago, United States) and MedCalc Statistical Software (version 14.8.1, Ostend, Belgium). Normality was assessed with the D’Agostino-Pearson test. Normally distributed continuous variables are presented as means ± standard deviations and were compared using a paired *t*-test. Non-normally distributed continuous variables are presented as median and inter-quartile ranges (IQR) and were compared using the Mann-Whitney U test. Categorical variables are presented as percentages and were compared using Fisher’s test. For statistical analysis, the LogMAR equivalent for counting fingers was 1.85, hand motion was 2.3, light perception was 2.8, and no light perception was 2.9 (Schulze-Bonsel et al., 2006).

## 3 Results

### 3.1 Demographics

This pilot study included 20 eyes from 20 patients (mean age 49.8 ± 20.0, 80% males) who underwent PACK-CXL high fluence treatment for infectious keratitis (Table 2). 17 (85%) patients had one bacterial infection; one patient (5%) was diagnosed solely with a fungal infection and two patients (10%) had a mixed infection—one with both fungal and bacterial infection, and the other had a positive culture for two distinct bacterial pathogens. The baseline clinical status right before PACK-CXL treatment is detailed in Table 2. Patients had a mean ulcer size of 5.7 ± 6.2 mm^2^ (median 3; IQR1.6–9.1). Ulcer stromal depth was estimated using a scale from 1 to 4, where 1 is infection restricted to the anterior stroma, and 4 is full thickness stromal involvement. The mean stromal involvement grade was 2.5 ± 1.3 (median 2.5; IQR:1–4). 50% of the patients had a central ulcer. Anterior chamber reaction was observed in 80% of cases and hypopyon in 30%.

**TABLE 2:** Patients’ demographics.

Age, mean ± SD	49.8 ± 20.0
Gender, male, n (%)	16 (80)
Eye, left, n (%)	11 (55)
Ethnicity, n (%)	Jewish: 15 (75)
	Bedouin: 5 (25)
DM, n (%)	3 (15)
Etiology, n (%)	Contact lens: 3 (15)
	Infected sutures: 3 (15)
	Trauma: 8 (40)
	Bullous keratopathy: 6 (30)
Central ulcer location, n (%)	10 (50)
Ulcer size, mm^2^, mean ± SD	5.7 ± 6.2
AC reaction, n (%)	4 (20)
Hypopyon, n (%)	6 (30)
Corneal melt, n (%)	3 (15)
Pathogen, n (%)	Bacterial solely: 17 (85)
	Fungi solely: 1 (5)
	Mixed: fungal and bacterial: 1 (5)
	Mixed: two distinct bacterial pathogens: 1 (5)
Topical medication, n (%)	Vancomycin: 16 (80)
	Ceftazidime: 16 (80)
	Moxifloxacin: 8 (40)
	Amphotericin: 3 (15)
	Ofloxacin: 2 (10)
	Natamycin: 1 (5)
Follow-Up periods in months, mean ± SD	1.7 ± 1.0
Baseline UCVA (logMAR), mean ± SD	1.57 ± 1.102
Baseline BCVA (logMAR), mean ± SD	1.40 ± 1.15
Final UCVA (logMAR), mean ± SD	1.31 ± 1.16
Final BCVA (logMAR), mean ± SD	1.21 ± 1.19

### 3.2 Ulcer and Re-epithelization

Prior to PACK-CXL, topical antibiotics were used for a mean of 1.6 ± 2.1 days (median 1; IQR: 0–2.5). Mean follow up time was 1.7 ± 1.0 (median 1; IQR: 1–2). No case had undergone corneal transplantation. Complete cornea re-epithelialization occurs at an average of 8.2 ± 2.8 days (range 2–14 days). The longest re-epithelization time of 14 days was observed in a diabetic patient with a full thickness bacterial ulcer. None of the patients were excluded from the study due to treatment failure.

### 3.3 Visual acuity and corneal endothelial cell density

**FIGURE 1:**
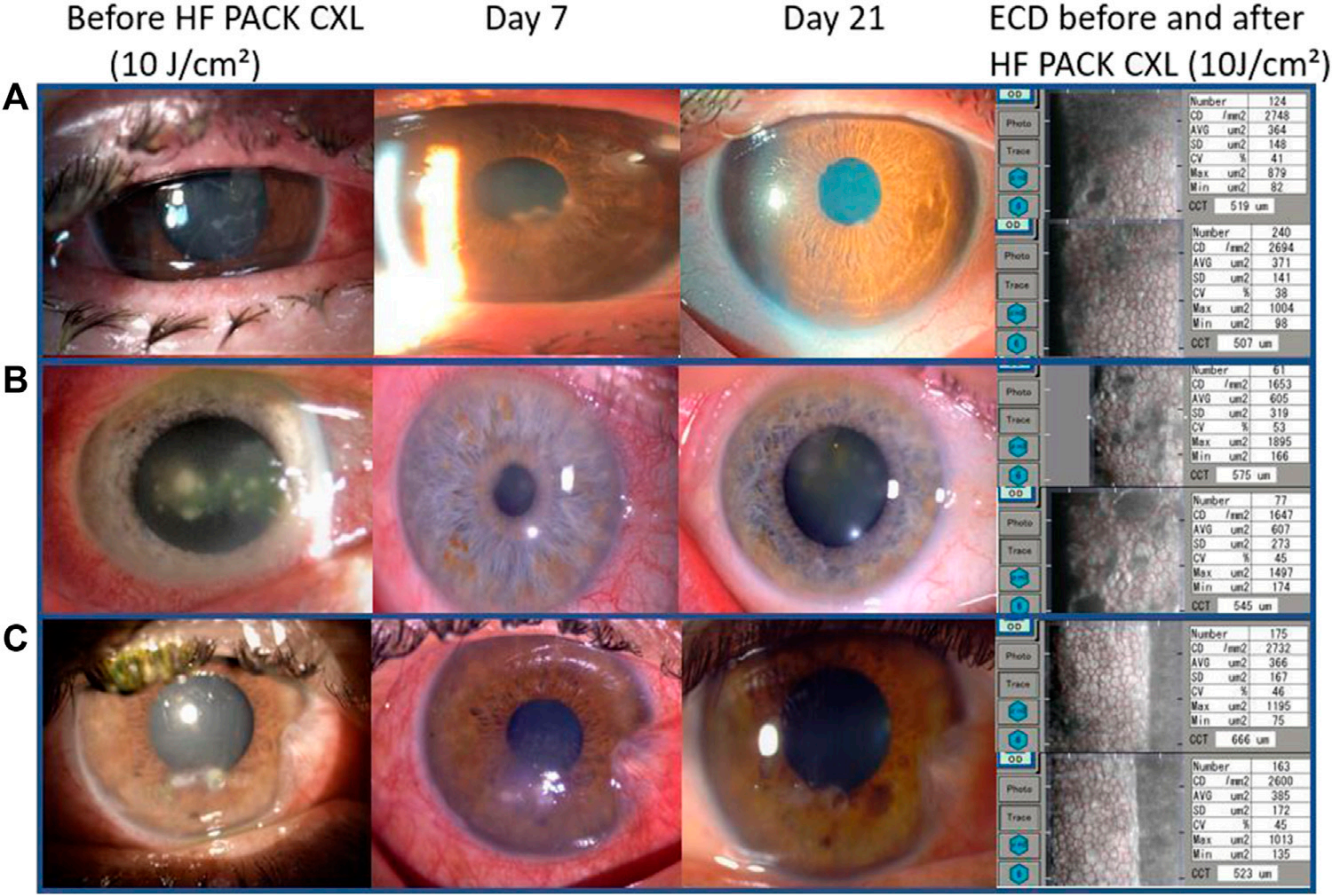
Slit-lamp images from three patients with mild to moderate keratitis. **(A)** *Fusarium solani* keratitis, **(B)** *Streptococcus pneumoniae* keratitis, and **(C)** *Klebsiella pneumoniae* keratitis. From left to right: at presentation, on day seven after treatment and 21 days after treatment with epithelial closure and a scar. The left column shows the endothelial cell density for each patient before (top) and 30 days after treatment (bottom).

Visual acuity, at the last follow-up visit, improved following PACK-CXL treatment (best-corrected visual acuity: mean difference 0.19 logMAR, 95CI: 0.04-0.33, *p* = 0.012); uncorrected visual acuity: mean difference 0.25 logMAR, 95% CI 0.06-0.44, *p* = 0.01). We compared endothelial cell density in nine patients where this data was available before and after PACK-CXL treatment (Figure 1). There was no significant change in endothelial cell density following treatment (2,562.1 ± 397.3 cells/mm^2^ vs. 2,564.8 ± 404.5, *p* = 0.96).

## 4 Discussion

This study reports on the outcomes of 20 eyes of 20 subjects who underwent high fluence PACK-CXL treatment for infectious keratitis at the slit lamp.

**FIGURE 2:**
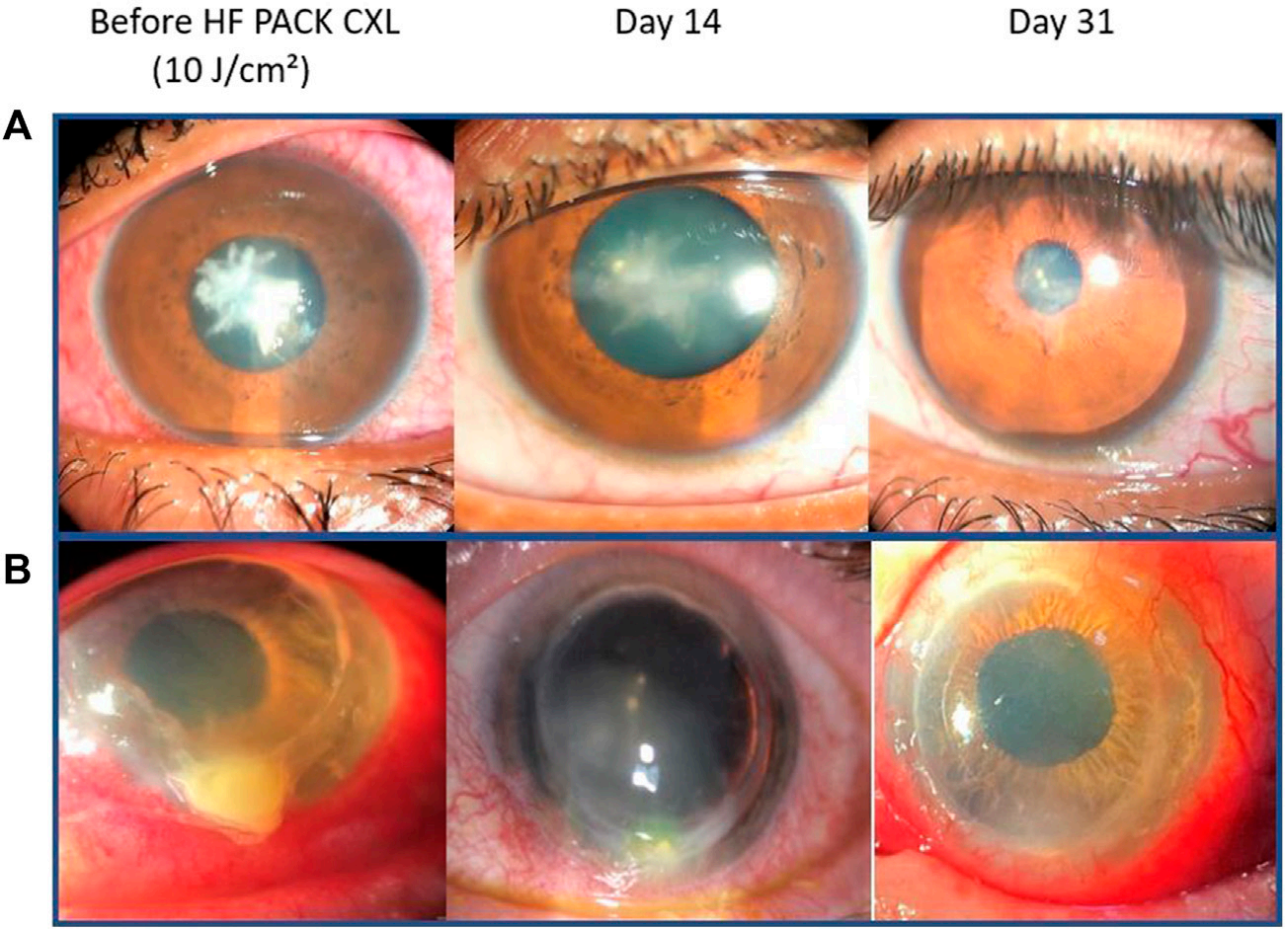
Slit-lamp images from two patients with severe keratitis. **(A)** *Streptococcus mitis* keratitis and **(B)** *Staphylococcus aureus* keratitis. From left to right: at presentation, at 14 days with almost completed epithelial closure and beginning of a scar formation, and 30 days after treatment with epithelial closure and corneal opacity.

We found the procedure to be effective, with re-epithelialization occurs at an average of 8.2 ± 2.8 days, the longest period being 14 days. Based on the observed epithelial closure within 14 days, it is consistent with a non-persistent corneal ulcer (HypothesisVaidyanathan et al., 2019). As a result, we can confidently conclude that PACK-CXL as an adjuvant therapy has been successful. Our results also show that it is a safe procedure, with no complications observed during the study, despite large ulcers and a severe degree of infection (Figure 2).

PACK-CXL is thought to act through three mechanisms (Price and Price, 2016): First, the chromophore’s planar ring structure intercalates between the DNA bases, thus limiting microbial reproduction; Second, reactive oxygen species are released, directly destroying pathogen cell walls; Finally, activated chromophores form covalent connections with the corneal stroma. This alters the collagen fibers' tertiary structure, reducing the number of collagenase binding sites and making them more resistant to enzymatic destruction. Importantly, these effects are unaffected by the level of pathogen antimicrobial drug resistance, which may be of particular value in, for example, cases of bacterial keratitis caused by highly antibiotic resistant strains.

In 2000, PACK-CXL treatment for melting corneal ulcers was used for the first time in a clinical setting. A single UV-radiation with two ultraviolet diodes and a wavelength of 370 nm with an energy of 2.5 mW/cm^2^ was administered to four patients suffering from melting ulcers of the cornea of various origins. The melting of the cornea ceased in three of the four individuals after treatment. A surgical operation could be postponed, at least temporarily (Irradiation of cornea, 2000).

Several protocols have been tried since then to optimize the effect, including the Dresden protocol developed for corneal ectasia. We have speculated that more energy is required to remove the infection effectively, particularly in deeper ulcers (Hafezi et al., 2020; Kling et al., 2020).

Our results are in line with previous studies. Awad et al. used rabbit models to test the efficiency of different PACK-CXL protocols on fungal keratitis, vs. Voriconazole as control. They found all protocols including the standard Voriconazole treatment to be effective with no statistically significant difference between them (Awad et al., 2022). Hafezi et al. compared antimicrobial therapy and PACK-CXL using of fluences of either 5.4 J/cm^2^ or 7.2 J/cm^2^ in a phase 3 trial that involved patients with infectious keratitis (Hafezi et al., 2022a). They found no significant difference between the groups in epithelial healing time. Of note, they used PACK-CXL as a stand-alone treatment, while in our study it was used as adjuvant to the standard of care. Pettersson et al. reported a case of a 24 years old woman with a severe *Acanthamoeba* keratitis, who was dramatically deteriorating despite an intensive drug regimen with adjuvant Dresden protocol PACK-CXL(24). The woman eventually got better only after high fluence PACK-CXL of 7.2 J/cm^2^, after which no further anti-amoebic treatment was needed. Another interesting case report of fungal keratitis was authored by Hafezi et al. (Hafezi et al., 2022b). They reported a 79 years old man who had a severe ulcer which did not improve with an aggressive intra-venous and topical anti-fungal treatment. He was then received one treatment of PACK-CXL with total fluence of 7.2 J/cm^2^ after which he got only slightly better. A week later it was decided to perform another high fluence PACK-CXL treatment, ultimately reaching a level of 14.4 J/cm^2^, fractionated across 2 doses. Substantial improvement was observed starting at day 1 after the second treatment. Knyazer et al. reported even greater levels of fluence given to their patient suffering from *Pseudomonas aeruginosa* ulcer (Knyazer et al., 2021). After further deterioration was noted in spite of standard topical treatment, the patient had been through two treatments of high fluence PACK-CXL, ultimately ending up receiving 21.6 j/cm^2^. 2 days after the last treatment, marked improvement was noted with final epithelial closure on day 6 after the procedure. The data presented in our study is, to our knowledge, the first pilot testing the therapeutic effect of high fluence PACK-CXL as an adjuvant therapy on a cohort of patients with infectious keratitis from various pathogens.

Another significant aspect of this study is that the PACK-CXL treatment was administered at the slit lamp. Cross-linking is typically a procedure performed with the patient in the supine position in the operating rooms (Hafezi et al., 2021), because irradiation time in the Dresden protocol is 30 min. However, In the setting of shorted irradiation time, such as in our protocol, the procedure can be performed at the slit lamp, allowing a more convenient, efficient, and accessible option for patients, while also maintaining the treatment’s effectiveness and safety.

The limitations of this study include the lack of a control group, short follow-up, and the small number of included patients. These limitations limit our ability to compare high fluence PACK-CXL to the standard of care and to other PACK-CXL protocols. However, we intended to show effectiveness and safety, superiority of the treatment was not one of the aims of this study. A large-scale, randomized, controlled study would be warranted to further strengthen our results and to assess the benefits of this protocol over the standard of care and over the use of lower fluence CXL protocols.

In conclusion, our study contributes valuable evidence to the growing body of knowledge that supports the advantages and efficacy of high fluence PACK-CXL. As infectious keratitis is a sight-threatening condition with often poor outcomes, we believe that the intensified treatment protocol with increased energy and exposure time in high fluence CXL has the potential to aid in the process of healing, providing a valuable therapeutic approach for this condition.

## Data Availability

The original contributions presented in the study are included in the article/Supplementary Material, further inquiries can be directed to the corresponding author.
